# Simultaneous Tunnel Grafting and Anterior Cruciate Ligament Reconstructions Revision Using Double Suspensory Fixation: A Single-Stage Solution

**DOI:** 10.1016/j.eats.2023.08.011

**Published:** 2023-12-04

**Authors:** Pouya Tabatabaei Irani, Mohammad Ayati Firoozabadi, Hesam Toofan, Seyed Mohammad Milad Seyedtabaei, Mohammad Poursalehian, Mohammadmahdi Ghasemian, Seyed Mohammad Javad Mortazavi

**Affiliations:** Joint Reconstruction Research Center, Orthopedic Surgery Department, Imam Khomeini Hospital, Tehran, Iran

## Abstract

The anterior cruciate ligament (ACL) is often vulnerable to sports-related injuries, leading to numerous ACL reconstructions (ACLRs) annually in the United States. Although largely successful, these procedures face the risk of recurrent instability due to graft failure. ACLR failures are typically attributed to technical errors and patient-related factors, with improper positioning of the tibial and femoral tunnels as the most common technical mistake. Current 2-stage revision techniques involve primary bone grafting followed by secondary tendon graft placement, resulting in increased costs and extended rehabilitation times. This article proposes a single-stage revision strategy involving simultaneous tunnel grafting and ACLR revision. The method employs double suspensory fixation by adjustable loop buttons, thereby eliminating the dependence on metaphyseal bone stock for stable graft fixation. This new procedure may offer a more efficient and cost-effective approach, reducing the need for a second surgery and potentially allowing patients to return to normal activities more quickly.

The anterior cruciate ligament (ACL), commonly susceptible to sports-related injuries, accounts for an estimated 100,000 to 200,000 annual ACL reconstructions (ACLRs) in the United States. While these reconstructions generally result in significant pain relief and improved knee stability (75%-95%), they are not without the potential for recurrent instability due to graft failure, affecting 0.7% to 10% of patients.^1^^,^^2^

Failures of ACLR can typically be attributed to 2 primary categories: technical errors and patient- or biology-related factors.^3^^,^^4^ Among technical errors, the most prevalent are improper positioning of the tibial and femoral tunnels, with such errors having significant impacts on metaphyseal bone defect.^2^ This necessitates the revision of ACLR techniques, which can significantly affect patients' treatment costs and rehabilitation.

Revision strategies must overcome multiple challenges, including achieving superior metaphyseal integration of the new graft, ensuring stable fixation, and facilitating a faster and more effective rehabilitation. Traditionally, 2-stage ACLR revision techniques have been employed to address these issues, involving primary bone grafting followed by secondary tendon graft placement, as detailed in Table 1.^3^^,^^5^^,^^6^ However, these methods often require patients to wait several months for the bone graft to unify before undergoing a second surgery, leading to increased costs, extended rehabilitation time, and the potential for further knee injuries due to prolonged instability.

**Table 1:** Previously Described Indications for Single- or Double-Stage ACLR Revision

Single-Stage ACLR Revision	Two-Stage ACLR Revision Surgery
Good range of motion	Stiff knee (>20° flexion deformity, <80° flexion)
Tunnel dilation <14-16 mm	Tunnel dilatation >14-16 mm
Completely incorrect or completely correct tunnel	Partially correct tunnel (two-thirds overlap with ideal position)
Smaller coronal plane deformity requiring correction (<10°)	Large coronal plane deformity requiring correction (>10°)
Posterior tibial slope ≤10°	Posterior tibial slope >12°

Adapted from Tapasvi S, Shekhar A. Revision ACL reconstruction: Principles and practice. *Indian J Orthop* 2021;55(2):263-275.

ACL, anterior cruciate ligament; ACLR, anterior cruciate ligament reconstruction.

In certain instances, the tunnels may even need to be overreamed and overwidened during the first stage to generate fresh metaphyseal bone for new graft integration, which could exacerbate metaphyseal loss. This potential for significant metaphyseal bone stock loss poses a risk to stable graft fixation, emphasizing the importance of the fixation method employed.

Various fixation methods for primary ACLR have been proposed, with Silva and Sampaio^7^ introducing the use of double suspensory fixation by adjustable loop buttons on both the tibia and femoral side. This technique holds the advantage of achieving cortical fixation in place of metaphyseal fixation, thereby eliminating the reliance on metaphyseal bone stock for stable graft fixation.^7^^,^^8^

In light of these considerations, we propose a technique involving a single stage of simultaneous tunnel grafting and ACLR revision. This method employs double suspensory fixation by 2 adjustable loop buttons, regardless of previous tunnel positions, size, and widening, and uses cortical fixation as an alternative to metaphyseal fixation (Video 1).

## Surgical Technique

### Patient Positioning

**Fig 1:**
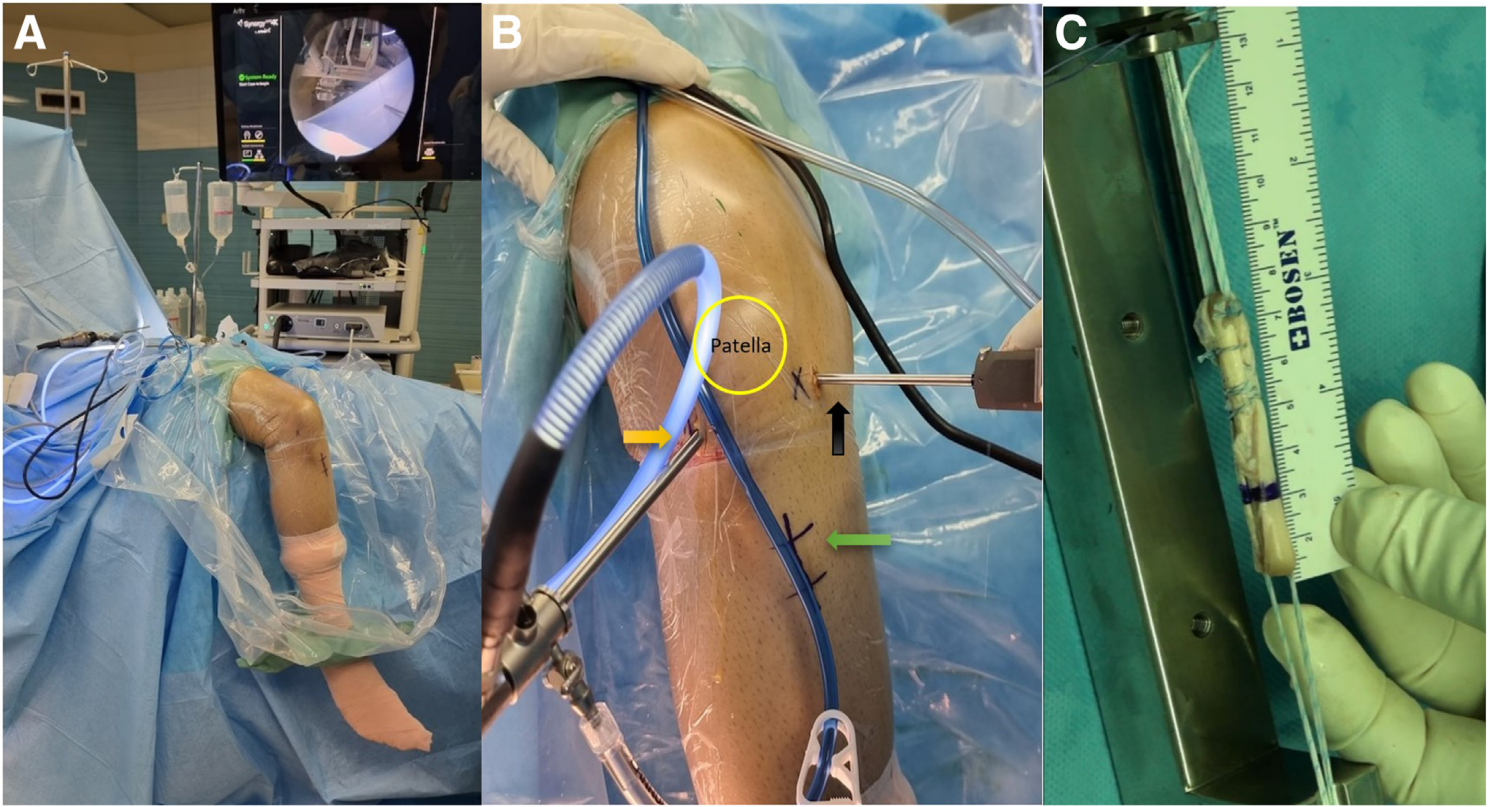
The initial steps of the knee surgery, performed with the patient in a supine position. (A) A full range of knee motion is facilitated by suspending the right leg. (B) The surgical area of the right leg is prepped, with the arthroscopic visual portal (indicated by a yellow arrow) and the working portal (black arrow) demarcated. Note the green arrow pointing to the scar from a previous anterior cruciate ligament reconstruction. (C) A quadruple semitendinosus allograft is meticulously prepared under tension and secured on both ends with Toggle Loc variable loop buttons.

The patient, after general or spinal anesthesia, is positioned supine on a radiolucent table. For a conducive surgical environment, the knee and calf are hung from the edge of the surgical table, maintaining a 90-degree flexion. This arrangement permits unrestricted knee movements and the potential for knee hyperflexion. Simultaneously, a tourniquet is applied to the proximal thigh (Fig 1A).

At this stage, prophylactic measures, including the administration of intravenous antibiotics, specifically 2 g cefazolin and 1 g tranexamic acid, are executed. The patient is then prepared for the surgical procedure, which involves skin prep and draping. Key points for anterolateral, medial, and anteromedial portal insertion are identified and marked on the prepared skin.

Subsequently, a sterile plastic sticking is applied to the surgical site. The tourniquet is then inflated, calibrated to the patient’s systolic blood pressure plus an additional 100 mm Hg, ensuring safe and efficient blood flow restriction.

Arthroscopic anteromedial working and anterolateral visual portals are then established. This setup is crucial to initially detect any coexisting knee joint lesions with the aid of a standard arthroscopic system (Fig 1B). Following this, the remnants of the failed ACL are arthroscopically debrided, which paves the way for an unobstructed field of view for the impending reconstruction procedure.

### Graft Preparation

In the event that a hamstring graft was not used in the previous surgery, it is now selected as the graft of choice. In contrast, if a hamstring graft was previously used, an allograft becomes the preferable option. The graft preparation and passage adhere to the methodology established by Silva and Sampaio.^7^ Specifically, a quadruple semitendinosus graft is prepared, to which 2 adjustable-length loop cortical buttons are attached on both ends. The devices employed are the ToggleLoc Device with ZipLoop Technology for the femur and the ToggleLoc XL Device with Inline ZipLoop Technology for the tibia, both produced by Zimmer Biomet. This prepared graft construct undergoes a tensioning process, where it is placed under 300 N of tension for 2 minutes. Before and after this tensioning, the length of the graft construct is meticulously measured. These measurements provide an essential comparison to ensure the graft's stability and integrity through the tensioning process (Fig 1C).

### Femoral Tunnel Preparation

The next phase of the procedure involves the preparation of the femoral tunnel. In the current case, the previous femoral tunnels are anatomically placed and exhibit no widening. The entrance of the previous femoral tunnel is identified via standard anteromedial and anterolateral portals. This tunnel is subsequently overdrilled using the inside-out transportal technique, implemented with a flexible No. 2 guidewire. It is important to note that this approach is applicable specifically to anatomically placed tunnels without widening. Other methods suitable for nonanatomic or near-anatomic femoral tunnels with widening exist, but these are not applicable to our current scenario.

The next step is the reaming of the femoral tunnel over the guidewire. This is performed using a 4.5-mm cannulated reamer (Zimmer Biomet), intended to remove any remnants in the tunnel. Following the removal of the guidewire, the tunnel is examined through the anteromedial portal. The goal of this examination is to detect any remaining soft tissue within the tunnel.

**Fig 2:**
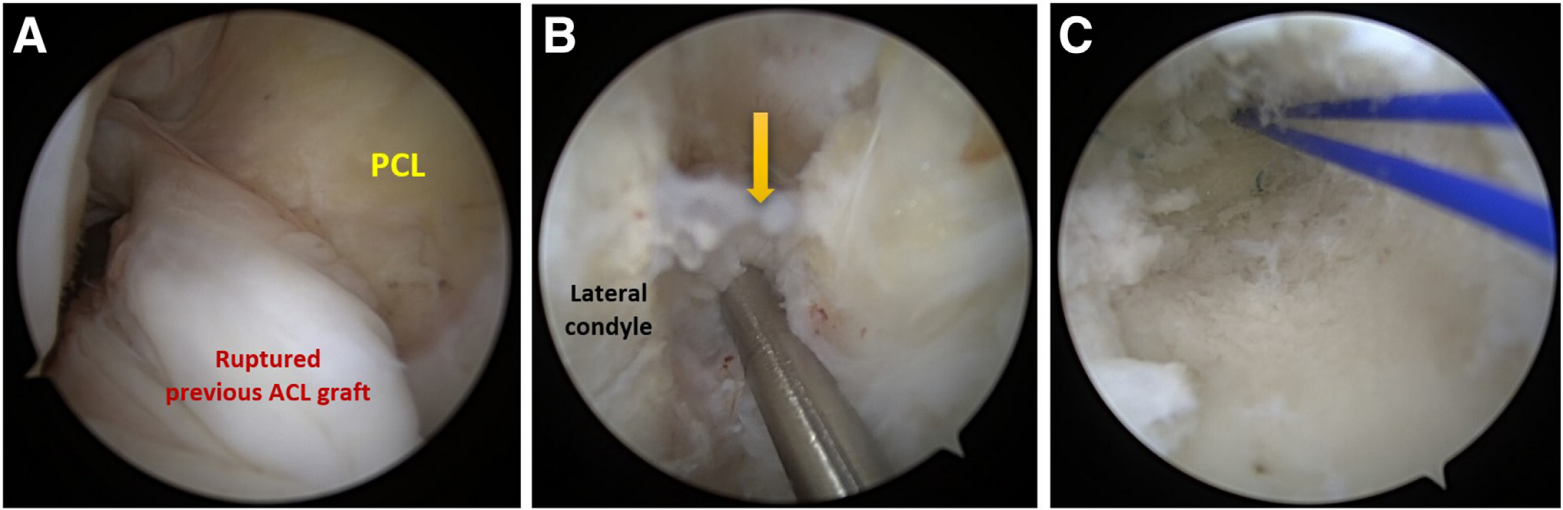
Highlighting the process of addressing a previously torn anterior cruciate ligament (ACL) graft in the right knee, visualized through the anterolateral portal. (A) Shows the damaged ACL graft. (B) A femoral tunnel guide wire is accurately placed at the previous tunnel entrance (marked by a yellow arrow). (C) After reaming and introducing a Nylon suture, the tunnel is carefully inspected through the medial portal to confirm the absence of additional soft tissue interference and to verify intact tunnel walls.

Simultaneously, the tunnel length is measured using an arthroscopic depth gauge. After these checks, the guidewire is repositioned within the tunnel. Customarily, a cannulated reamer (Zimmer Biomet) of the same size as the graft diameter is used to ream an equal length of the previous femoral tunnel. The final step in this phase of the procedure involves the placement of a shuttle suture from the lateral thigh and the anteromedial portal into the femoral tunnel (Fig 2).

### Tibial Tunnel Preparation

A suitable location for the new tunnel in the tibial bone is identified. Should the preexisting screw interfere with the intended tunnel position, it would be removed using a specialized surgical screwdriver. However, if no interference is present, the previous screw would be left undisturbed and a new tunnel created separately. This approach minimizes potential damage to the tibial bone compared to routinely removing the previous screw and typically yields superior fixation.

**Fig 3:**
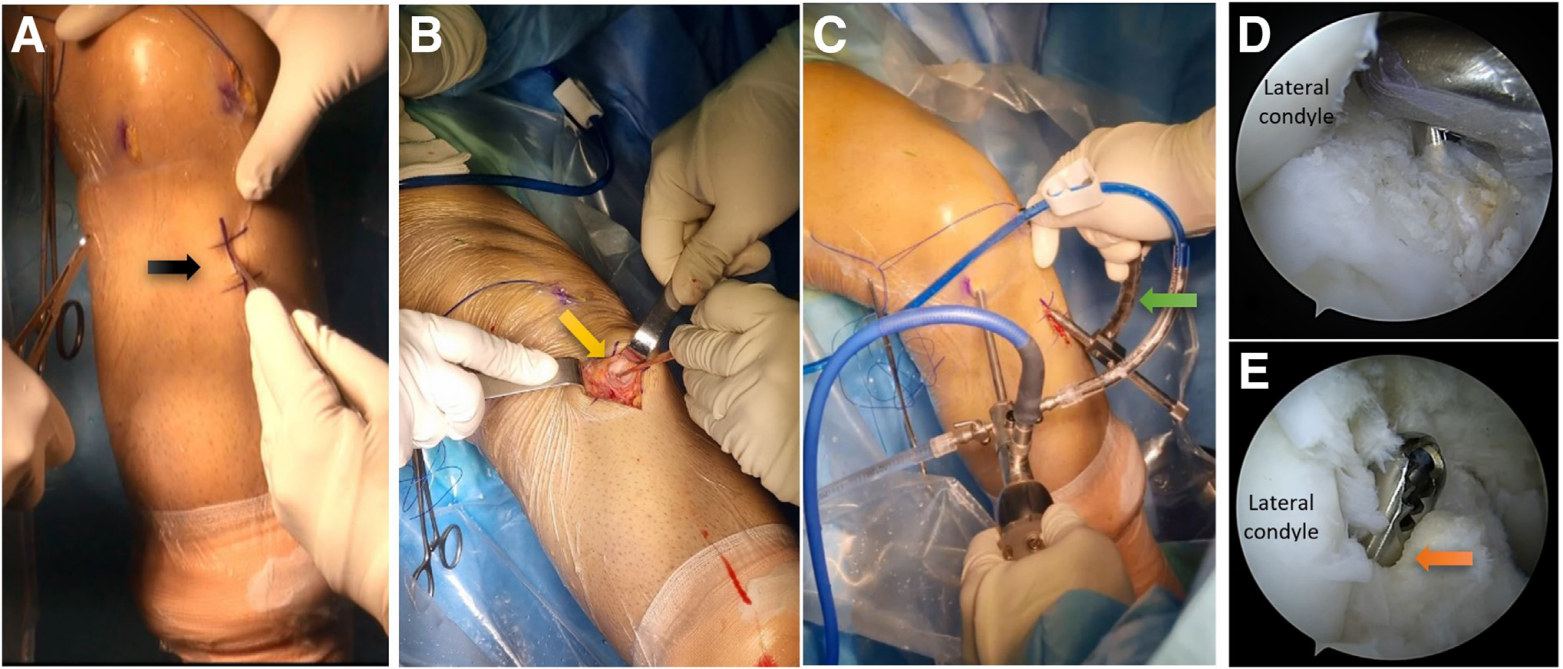
The process of creating a tibial incision on the right leg, in alignment with a prior anterior cruciate ligament (ACL) reconstruction incision. (A) A black arrow points to the previous incision. (B) The original tibial tunnel entrance is located and pointed out by forceps (yellow arrow). (C) The tibial tunnel guide (green arrow) is set at a 55° angle and positioned at the previous tunnel entrance on the tibial cortex. (D) The ACL footprint guides the proper positioning, visualized through the anterolateral portal. (E) The tibial tunnel (orange arrow) is thoroughly debrided with a shaver to remove any remaining soft tissue.

Following this, under arthroscopic visualization, the guidewire is positioned on the anatomic tibial ACL footprint. This enables the reaming process to remove any remnants in the previous tibial tunnel. Last, the tunnel is examined through the visual portal to detect any remaining soft tissue. This thorough inspection is critical to ensure the tunnel is sufficiently cleared and ready for the next steps in the procedure (Fig 3).

### Graft Passage and Fixation

After the femoral and tibial tunnels have been adequately prepared, the next step involves graft passage and fixation. Initially, the proximal tip of the graft, the reamed depth of the femoral tunnel, and the intra-articular part of the graft (typically 25-30 mm) are marked correspondingly. These markings serve as essential guides during the graft insertion.

**Fig 4:**
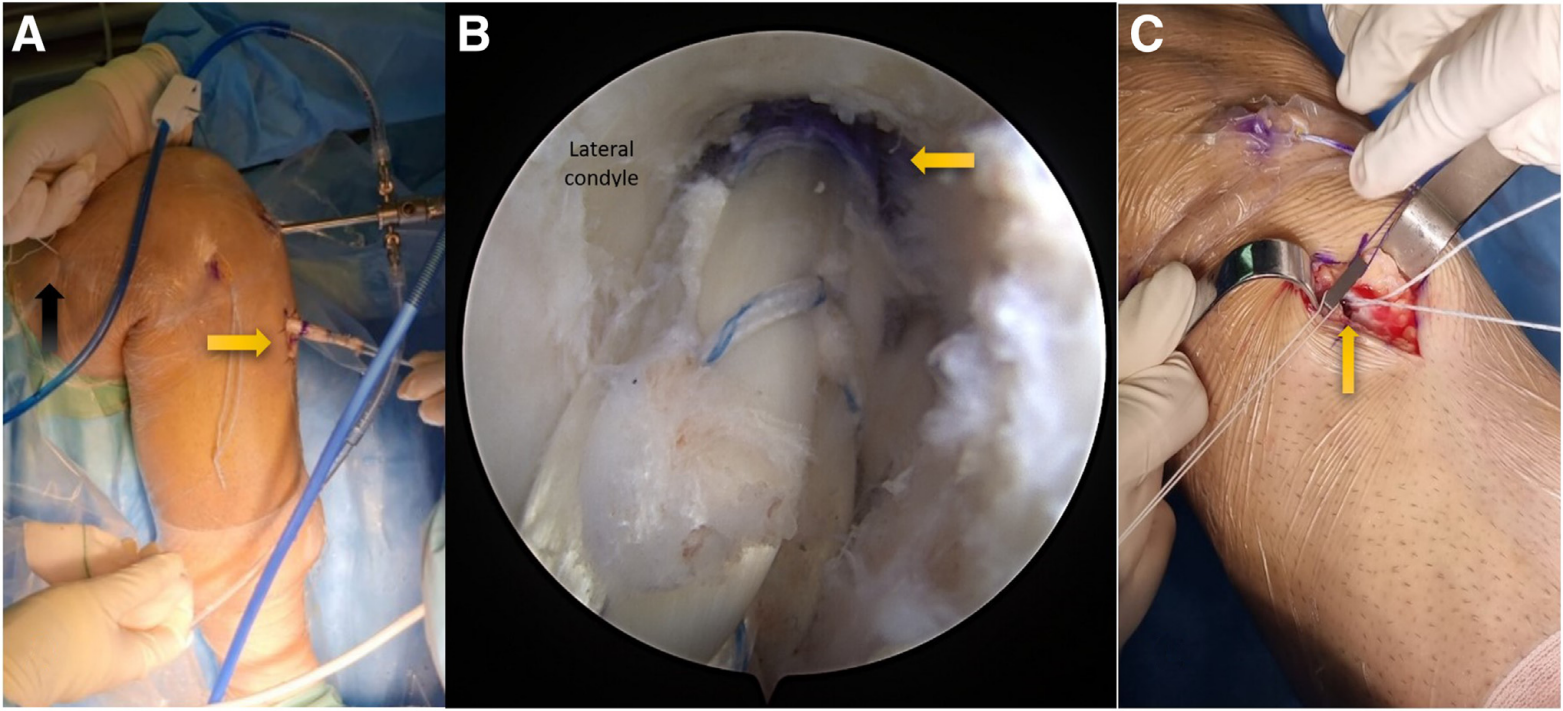
Depicting the placement of a new anterior cruciate ligament allograft (yellow arrow) in the right knee. (A) The allograft is pulled through the tunnels by tensioning the femoral side loop (black arrow). (B) The allograft is threaded through the femoral tunnel up to a premarked length (yellow arrow). (C) The tibial button is subsequently tensioned to secure its seating at the tibial cortex. Notice the tibial tunnel hole (yellow arrow) situated beneath the button.

The femoral adjustable graft loop is then inserted into the femoral tunnel and drawn through the tibial tunnel. This procedure is performed under direct arthroscopic visualization. As the first mark on the graft loop reaches the tunnel aperture, it signifies that the button has successfully navigated the femoral cortex proximally and is primed for flipping (Fig 4A).

Once the button flips, the graft is retracted to secure a solid femoral fixation. Subsequently, the femoral pull suture is exerted to draw the graft into the femoral tunnel until it aligns with the second marking (Fig 4B).

Following this, tension is applied to the tibial tensioning sutures, facilitating the seating of the graft and the button within the tibial tunnel and the cortical tibial bone, respectively. Last, the knee is moved through its range of motion 30 times to ensure graft flexibility. This cycling is followed by retensioning the graft. The retensioning process is accomplished by pulling the tibial sutures while the knee is in full extension, ensuring optimal graft function and stability (Fig 4C).

### Restoration of Tibial Bone Defect

**Fig 5:**
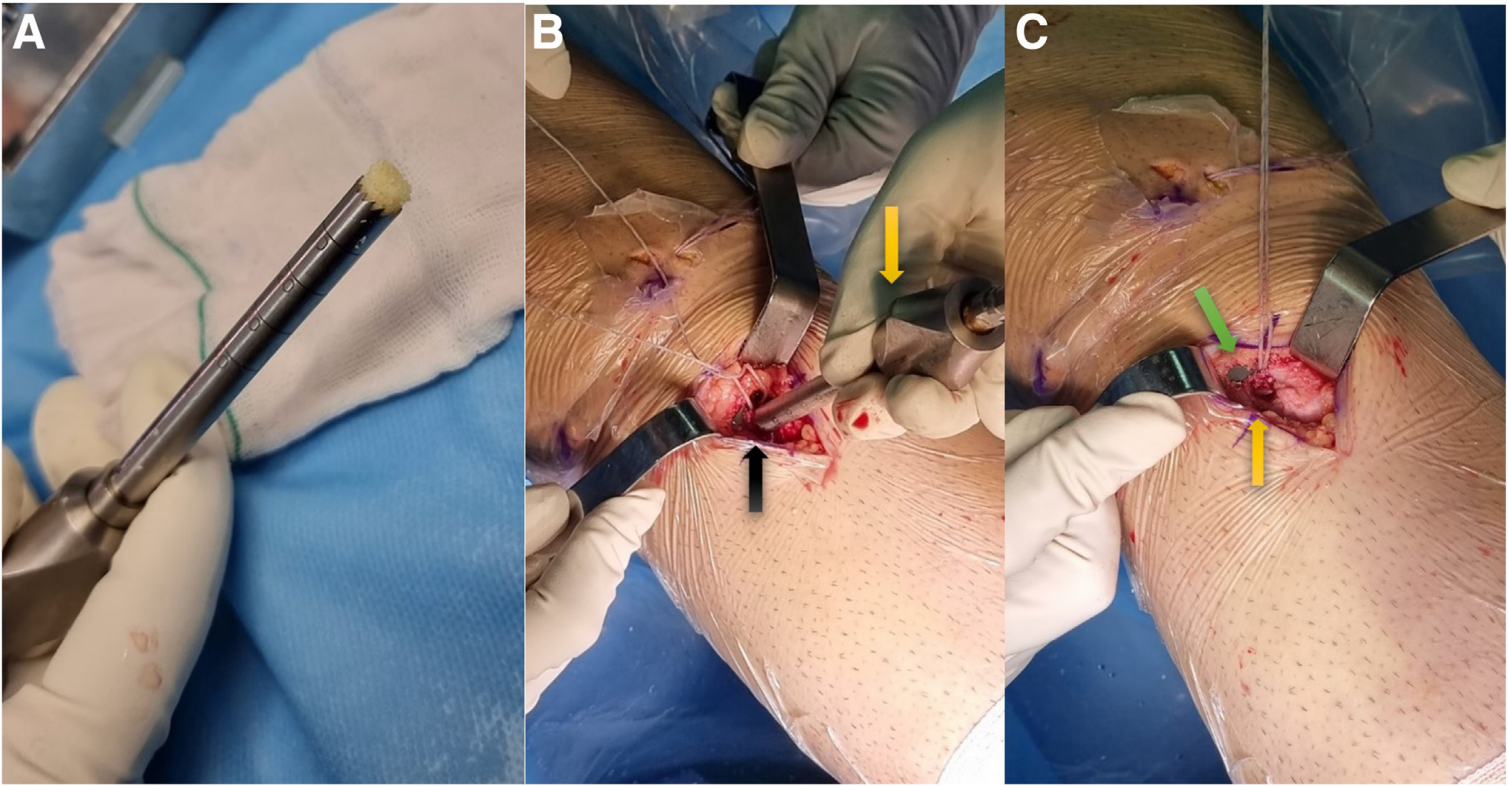
The preparation and placement of a cylindrical bone graft. (A) Bone cubes are impacted into the harvesting tube to produce a graft matching the tunnel size. (B) The harvesting tube (yellow arrow) is used to introduce the bone graft into the tibial tunnel (black arrow). (C) The button is adequately seated on the tibial cortex (green arrow) and the tibial tunnel is entirely filled with bone grafts (yellow arrow).

The final phase of the procedure involves the restoration of the tibial bone defect. Initially, the tunnel is filled with a cylindrical cancellous bone allograft. This allograft is carefully prepared and compacted using a properly sized bone harvester device (Zimmer Biomet). The allograft is crafted to be slightly shorter than the tibial tunnel length to prevent joint penetration (Fig 5).

Subsequently, the stability of the fixation is confirmed by assessing the knee range of motion. Once deemed satisfactory, the joint is thoroughly irrigated. The surgical wounds are then closed using monofilament absorbable sutures. Detailed descriptions of the procedural pearls, pitfalls, and advantages and disadvantages can be found in Tables 2 and 3, respectively.

**Table 2:** Pearls and Pitfalls

Pearls	Pitfalls
It is crucial for the surgeon to have the ability to move the knee from full extension to hyperflexion during the procedure.	As the ToggleLoc ZipLoop lacks a string for flipping the button, the button flip technique cannot be used during this procedure. Instead, the proximal button's placement within the femoral tunnel can be monitored through the far medial portal to ensure it will rest on the lateral femoral cortex.
Identification of the precise anatomic origin and insertion points of the ACL within the knee is critical.	Advising the patient to cease smoking or the use of nicotine products is necessary to prevent tibial nonunion.
Identification and appropriate treatment of any concomitant ligament injuries should be prioritized.	Care must be taken to prevent the intra-articular entrance of bone graft during taping.
If necessary, ALL tenodesis should be performed at the end of ACLR revision surgery.	

ACL, anterior cruciate ligament; ACLR, anterior cruciate ligament reconstruction; ALL, anterolateral ligament.

**Table 3:** Advantages and Disadvantages

Advantages	Disadvantages
Single-stage procedure needed only	More technical demanding
Less metaphyseal bone loss by not overreaming tunnels	High learning curve
Better ACL graft incorporation after filling the tunnel with bone graft	Loosening of the adjustable loop in comparison to the fixed loop following cyclic loading in biomechanical studies
Preserving the tibia bone stock for potential later procedures	
More stable graft fixation using cortical button instead of metaphyseal fixation	
Less cost and rapid rehabilitation because of having single procedure	

ACL, anterior cruciate ligament; ACLR, anterior cruciate ligament reconstruction.

**Fig 6:**
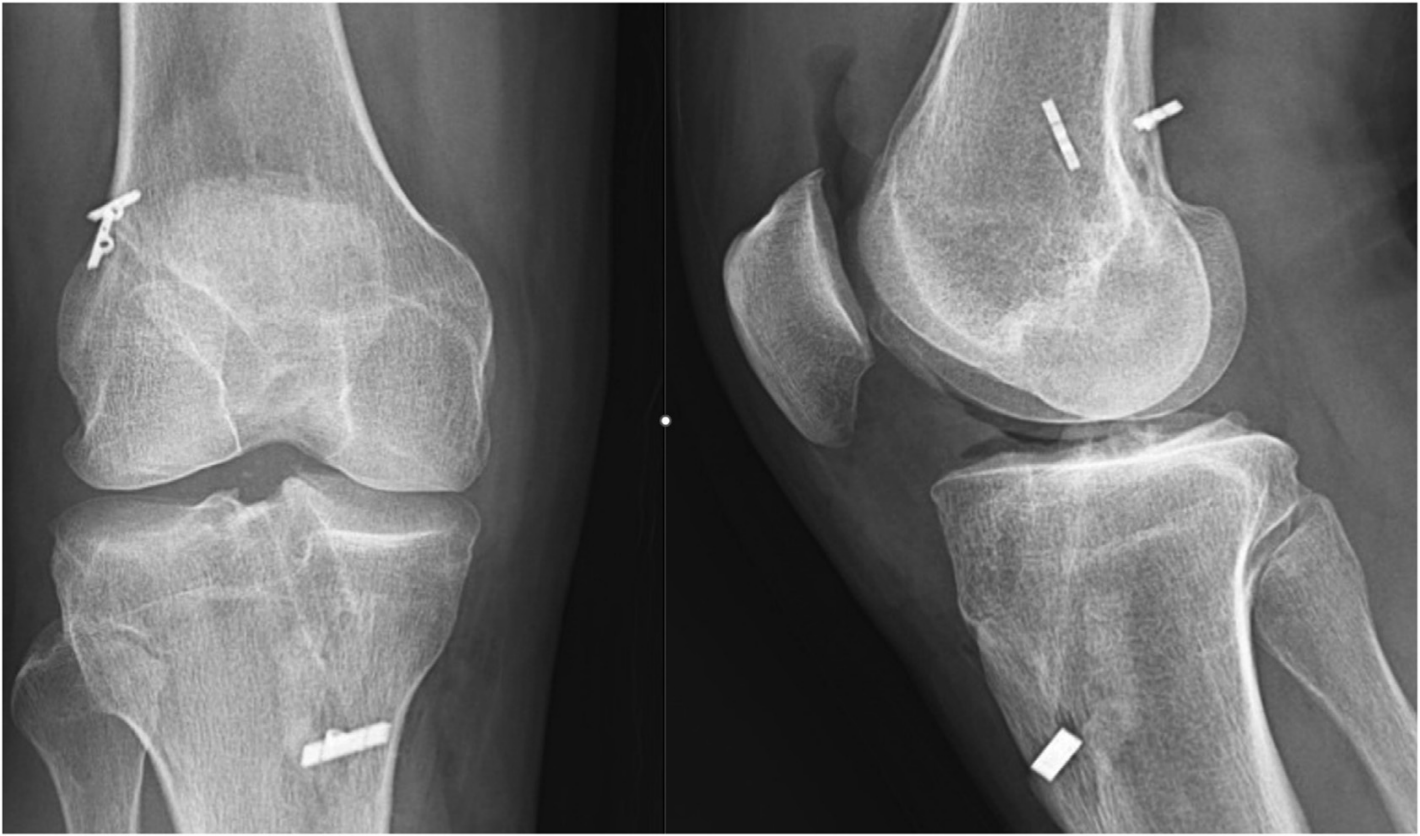
A postoperative radiograph of the right knee showcasing the results of a single-stage anterior cruciate ligament reconstruction revision with double suspensory fixation and simultaneous tunnel grafting.

Following the surgical procedure, a postoperative radiographic evaluation is performed to verify the optimal positioning of the newly created tunnels. Additionally, the placement of the button device is examined using x-ray to confirm its appropriate location (Fig 6). In terms of postoperative care, there is no requirement for knee immobilization. An active weightbearing, closed-chain range of motion is initiated the day after surgery, followed by an open-chain range of motion after 3 weeks. The patient is typically discharged the following day, marking the successful completion of the procedure.

## Discussion

ACLR has a success rate of 75% to 97%. However, due to this range, thousands of ACLR revisions occur annually, and unfortunately, these have inferior outcomes compared to primary reconstructions.^9^ According to a study by Jaecker et al.^10^ on 167 failed ACLRs, technical error, trauma, and biological failure accounted for 64.5%, 29.1%, and 6.4% of cases, respectively.^4^

There are a few techniques for ACLR revisions, each considering the position and widening of previous femoral and tibial tunnels, which are common complications. Some single-stage methods might necessitate overreaming the tunnel to create fresh walls, even when not dilated, resulting in more metaphyseal bone defect. To address this issue, solutions include grafting the previous tunnel and making new ones,^11^, ^12^, ^13^ utilizing larger screw fixation, using bone from the graft (Achilles tendon allograft with bone or semitendinosus autograft with bone),^14^ occupying the bone void with stacked screw technique,^15^ or making an “over-the-top” type of femoral tunnel to avoid tunnel overlap.^16^ However, these solutions may not always yield superior metaphyseal fixation and outcomes.^3^^,^^17^ Precise attention and case-based decision-making are needed when overlapping new tunnels with previous ones or when dealing with tunnel widening.

One of the primary challenges during ACLR revisions is the careful consideration of the position of previous tunnels and whether they interfere with the new graft fixation.^18^ Another concern is tunnel widening, which makes it difficult to maintain metaphyseal fixation of the allograft. In the case of tunnel widening of less than 14 to 16 mm, a single-stage revision is usually performed with overdrilling tunnels to a larger diameter. This method can result in more metaphyseal bone loss and require a larger diameter graft. Two-stage revisions, usually employed when tunnel widening is >14 to 16 mm, start with initial tunnel debris removal and grafting, followed by graft fixation if adequate bone union is found on a computed tomography scan after 4 to 6 weeks.^1^^,^^3^^,^^19^

However, 2-stage procedures increase hospitalization costs, require more operations, and lengthen rehabilitation periods. In this context, we have employed the double suspensory ACLR method, as described by Silva and Sampaio,^7^ in a single-stage procedure. This method can be used regardless of the tibial and femoral tunnel positioning and tunnel widening.

In this technique, the tibial and femoral tunnels are drilled either on the previously near-anatomic tunnels or new ones. The graft fixation is achieved through 2 adjustable buttons, which makes it easier to address the metaphysical bone defect in the case of tunnel widening, while not requiring overreaming of the tunnel to larger diameters. Filling the tunnel with a bone allograft also aids in rapid bone integration, preserves bone for potential future revisions, and reduces metaphyseal bone loss. Therefore, this approach minimizes the need for a second-stage surgery and reduces hospitalization costs and rehabilitation time, allowing the patient to return to normal life more quickly.

## Supplementary Data

ICMJE author disclosure forms

Video 1 A detailed demonstration of our surgical technique for tunnel reaming and graft insertion for anterior cruciate ligament reconstruction. The patient is placed in the supine position and prepped with a tourniquet and intravenous antibiotics. Key locations for various portal insertions are marked and the surgical site is prepared. This is followed by careful graft selection and preparation, including attaching adjustable-length loop cortical buttons to both ends of the graft and tensioning. The arthroscopic technique is used to identify, overdrill, and ream the femoral tunnel. Attention is then directed to the tibial bone to select a new tunnel location, which is then reamed and thoroughly cleared of any remnants from the previous tibial tunnel. The graft is carefully inserted through both femoral and tibial tunnels, showing secure fixation. The final steps involve restoring the tibial bone defect with a cylindrical cancellous bone allograft, checking the stability of the fixation, and closing the wound using monofilament absorbable sutures.
